# Comparative analysis of MitraClip/TriClip and PASCAL in transcatheter tricuspid valve repair for tricuspid regurgitation: a systematic review and meta-analysis

**DOI:** 10.1186/s12872-024-04201-6

**Published:** 2024-10-14

**Authors:** Mahmoud Balata, Mohamed Ibrahim Gbreel, Marwa Hassan, Marc Ulrich Becher

**Affiliations:** 1 University of Erlangen-Nuremberg, Erlangen, Germany; 2 University of Bonn, Bonn, Germany; 3 Faculty of Medicine, October 6 University, Giza, Egypt; 4 Theodor Bilharz Research Institute, Giza, Egypt; 5 Department of Cardiology, City hospital Solingen, Solingen, Germany

**Keywords:** Tricuspid regurgitation, TriClip, MitraClip, Pascal, Transcatheter tricuspid valve repair, Meta-analysis

## Abstract

**Background:**

The edge-to-edge transcatheter tricuspid valve repair (TTVR) has emerged as a promising technique for the treatment of tricuspid regurgitation (TR). Despite its potential, comparative data on the performance of the novel edge-to-edge devices—MitraClip, PASCAL, and TriClip—remain controversial. In this study, we aim to evaluate the safety and efficacy of these devices in treating TR.

**Methods:**

Five databases were systematically searched up to May 2023, with an updated search conducted in May 2024. Only original studies were included in the analysis and were critically evaluated using an adapted version of the Newcastle-Ottawa Scale (NOS) for observational cohort studies and the Cochrane Risk of Bias (ROB) tool for randomized controlled trials.

**Results:**

The database search yielded 2239 studies, out of which 21 studies were included in the final analysis. These studies encompassed a total of 2178 patients who underwent TTVR using either the MitraClip, TriClip, or PASCAL devices. The risk of bias across these studies ranged from moderate to high. No significant differences were found among the three devices in terms of effective regurgitant orifice area (EROA) and tricuspid regurgitant volume. However, TriClip demonstrated statistically superior efficacy in reducing vena contracta compared to both MitraClip and PASCAL (*P* < 0.01) [TriClip: (MD = -7.4; 95% CI: -9.24, -5.56), MitraClip: (MD = -4.04; 95% CI: -5.03, -3.05), and PASCAL: (MD = -6.56; 95% CI: -7.76, -5.35)]. The procedural success rates and incidence of single leaflet device attachment (SLDA) were similar across all devices. Furthermore, there were no significant differences in mortality, stroke rates, or major bleeding events among the three devices.

**Conclusion:**

The TriClip outperforms the MitraClip and PASCAL in reducing vena contracta width, indicating greater effectiveness for severe tricuspid regurgitation. All devices show similar safety profiles and procedural success rates. Further research is needed to confirm these results.

**Supplementary Information:**

The online version contains supplementary material available at 10.1186/s12872-024-04201-6.

## Introduction

Tricuspid regurgitation (TR) is a prevalent valvular disease associated with worsening heart failure symptoms and an elevated risk of mortality and morbidity [1, 2]. Despite tricuspid valve surgery being the conventional first-line therapy for TR, it poses a substantial peri-procedural mortality risk and may not yield significant clinical improvements [3, 4]. In contrast, transcatheter techniques emerge as a promising alternative for reducing TR in high-surgical-risk patients [5]. These approaches not only enhance symptoms and quality of life compared to medical therapy but also maintain a low peri-procedural mortality rate [6].

Various edge-to-edge transcatheter tricuspid valve repair (TTVR) techniques, including MitraClip and TriClip (both by Abbott, Santa Clara, California, USA), as well as the PASCAL transcatheter valve repair system (Edwards Lifesciences, Irvine, California, USA), have shown promising results [7–9]. However, the lack of a comparative analysis among these devices highlights the need for a comprehensive meta-analysis. This analysis should evaluate the safety and effectiveness of these devices for treating TR, helping clinicians in making informed choices about the optimal device for TTVR in patients with TR.

## Materials and methods

The current study was conducted following the approaches outlined in the Cochrane Handbook for Systematic Reviews of Interventions [10]. Throughout the drafting of the manuscript, strict adherence to the Preferred Reporting Items for Systematic Reviews and Meta-Analyses (PRISMA) statement guidelines was maintained [11].

### Search strategy

The following electronic databases were systematically searched up to May 2023: PubMed, Web of Science (WOS), Scopus, Medline, and Cochrane. In addition, we updated the search in the similar databases in May 2024. The search strategy used the following search terms: (Tricuspid) AND (Insufficiency OR Incompetence OR Regurgitation) AND (MitraClip OR Pascal OR TriClip). References from all included studies were screened to ensure no studies were missed and to guarantee high-quality screening.

### Eligibility criteria

Studies were included based on the following eligibility criteria: patients undergoing transcatheter tricuspid valve repair (TTVR) with interventions MitraClip, Pascal, or TriClip, and study designs that were either single-arm clinical trials or cohorts. Conversely, we excluded non-human studies, conference abstracts, cohorts, case series, case-control studies, case reports, and studies not in English.

### Screening and study selection

Using Zotero software, [12] we compiled records from various databases and eliminated duplicates. The retrieved references underwent screening to assess relevance. This screening process was divided into two steps: first, title and abstract screening, followed by full-text review to determine final eligibility, utilizing Microsoft Excel Spreadsheets [13]. At least two independent authors conducted each step, comparing findings. Any disagreements were resolved through group discussions.

### Quality assessment

For all single-arm clinical trials included, the quality was evaluated using the ROBINS-I Cochrane Collaboration tool [14]. This evaluation covers several domains: bias due to confounding, bias in selection of participants for the study, bias in classification of interventions, bias due to deviations from intended interventions, bias due to missing data, bias in measurement of outcomes, and bias in selection of the reported result. The assessment determines whether there is a low, high, or unclear risk of bias. For cohort studies, the Newcastle-Ottawa Scale (NOS) was used [15]. It encompasses the following domains: Sample selection criteria, Comparability and Exposure.

### Data extraction

Two independent authors extracted data from the included studies, covering the following aspects: study design, setting, sample size, follow-up duration, protocol registration, population definition, primary outcome measures, and baseline characteristics.

### Primary and secondary endpoints

The primary endpoints included Vena contracta width (mm), effective regurgitant orifice area (EROA)(mm^2^), tricuspid regurgitant volume (ml), Tricuspid annulus diameter (mm), Tricuspid annular plane systolic excursion (TAPSE) (mm), right ventricular fractional area change (%), Systolic pulmonary artery pressure (mm/Hg), left ventricular ejection fraction (%), six-minute walking test (6-MWT), degree of regurgitation after the procedure, and New York Heart Association (NYHA) classification.

The secondary outcomes were Procedural success, Procedure time (min), Fluoroscopy duration (min), Length of hospital stay (days), ICU stay (day), 30-day Mortality, In hospital Mortality, Myocardial infarction, Stroke, Major bleeding, Acute kidney injury, Tamponade, Conversion to surgery, and Single-leaflet device attachment.

### Statistical analysis

We used R version 4.2.2 (2022-10-31) and R Studio version 2022.07.2 (2009–2022) RStudio, Inc.). For dichotomous data, we analyzed the risk ratio (RR) and 95% confidence interval (CI), while continuous data were analyzed as mean difference (MD) and 95% CI. To assess statistical heterogeneity among studies, we conducted a visual inspection of the forest plot, in addition to using the I-squared (I^2) and chi-squared (Chi^2) statistics. I^2 values of 50% or higher were considered indicative of significant heterogeneity. When there was significant variation in the data, a random-effects model was employed; otherwise, a fixed-effect model was applied.

## Results

### Study selection

**Fig. 1:**
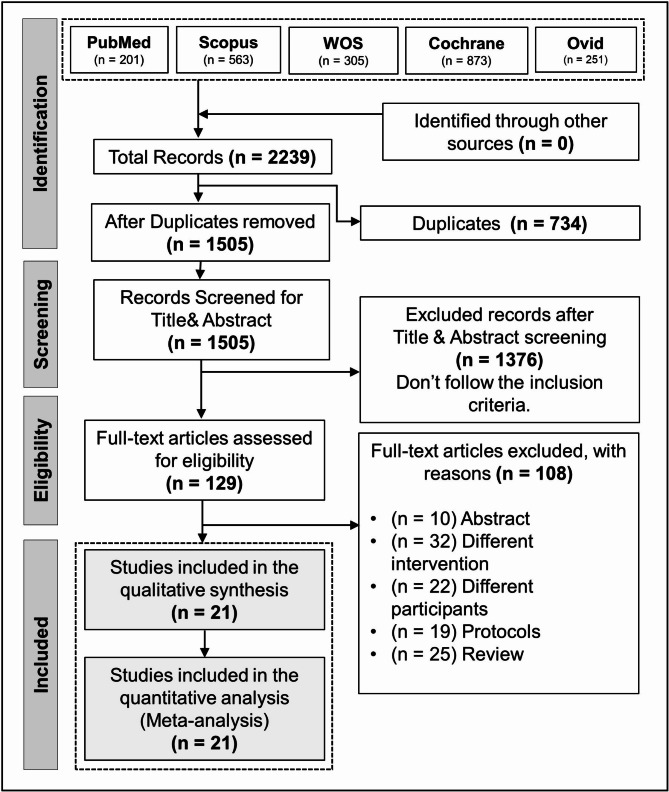
PRISMA flow diagram of the literature search results, illustrating the process of study selection and inclusion criteria

The comprehensive search across all databases yielded 2239 articles. Once duplicates were removed, 1505 articles were left for screening. After the initial screening, 129 articles were eligible for further evaluation. Following this, a secondary screening of these articles’ full texts resulted in 21 articles [7, 16–35] being selected for inclusion in the study and subsequent analysis (Fig. 1: PRISMA flow diagram).

### Characteristics of the included studies

Table 1 provides a summary of the included studies, which together involve a total of 2178 patients. Detailed characteristics of the participants are presented in Supplementary 2 Table S1.

**Table 1:** Summary of the included studies

Study ID	Year	Setting	Study Design	Sample Size	Follow-up (days), mean (SD)	Population definition	Primary outcome measures	No. of Clips
Mahowald et al.	2021	Rochester, Minnesota	Retrospective Cohort	38	339 (125.25)	Patients who underwent tricuspid TEER for at least moderate-severe TR alone or in conjunction with mitral TEER using the MitraClip.	Mortality, heart failure hospitalization, reintervention	NA
Low et al.	2021	Germany	Retrospective Cohort	120	30	Patients who treated for symptomatic isolated severe TR using the edge-to-edge repair technique.	TR severity, (NYHA) functional class, major adverse cardiac, cerebrovascular events	2 ± 1.7
Mehr et al.	2019	Germany	Retrospective Cohort	249	274 (187)	All patients included were symptomatic, with signs of right-sided or global heart failure. The majority of patients were in NYHA functional class III or IV and were deemed at high or prohibitive risk for surgery.	All-cause mortality, Unplanned rehospitalization for heart failure within 12 months after the procedure	0 (3.6), 1 (27.3%), 2 (43.8%), 3 (20.5%), 4 (4.4%), 5 (0.4%)
Nickenig et al.	2017	Germany	Multi-Center International single-arm trial	64	14 (18)	Patients with severe TR were recruited from 10 international centers. All patients were considered unsuitable fo surgery, and the interventional approach with the MitraClip system was offered as compassionate use. Patients were symptomatic with moderate to massive TR on optimal medical treatment.	TR was reduction by at least 1 grade, Effective regurgitant orifice area, vena contracta width, Regurgitant volume, Postprocedural Events, major vascular complications, in-hospital deaths, New York Heart Association class improvement,6-minute walking distance.	0 (3%), 1 (48%), 2 (23%), 3 (17%), 4 (2%)
Nickenig et al.	2019	Europe and the USA	Multicentre study prospective single-arm study	85	180	Patients with moderate or greater triscuspid regurgitation, New York Heart Association class II or higher, and who were adequately treated per applicable standards.	Reduction in tricuspid regurgitation severity by at least one grade at 30 days post procedure, The primary safety endpoint was a composite of major adverse events at 6 months.	NA
Ohno et al.	y d	Italy	Retrospective Cohort	146	660 (494)	Patients with symptoms or signs of left ventricle deterioration and 3 + or 4 + MR determined by combined transthoracic and transoesophageal echocardiogram considered to be at high-surgical risk by an interdisciplinary team underwent percutaneous edge-to-edge mitral valve repair with MitraClip.	The primary safety endpoint was the incidence of major adverse events at 30-day, he primary efficacy endpoint was freedom from death, surgery for mitral valve dysfunction, or grade ≥ 3 + MR at the 12-month follow-up after clip implantation.	NA
Orban et al.	2018	Germany	Dual centre single arm Cohort Study	50	188.7 (22.1)	All patients had clinical signs of right-sided HF. Patients were in New York Heart Association (NYHA) functional class III or IV despite optimal medical therapy and were deemed at prohibitive surgical risk by an interdisciplinary heart team.	The primary objective was to define the mid-term device safety and durability in TR reduction after 6 months.	NA
Otto et al.	2021	Germany	Retrospective Cohort	20	30	All patients undergoing transcatheter TV repair.	The safety and feasibility of the procedure, reduction of TR-grade.	1.8 ± 0.8
Toyama et al.	2017	USA	Multi-Center retrospective Cohort	102	365	All eligible patients were candidates for MV surgery for moderate–to-severe or severe chronic mitral regurgitation (MR). Symptomatic patients were required to have a left ventricular ejection fraction (LVEF) of > 25% and a left ventricular (LV) end-systolic diameter of ≤ 55 mm.	systolic pulmonary artery pressure (sPAP), Right-sided cardiac reverse remodeling, right ventricular fractional area change.	NA
Ruf et al.	2021	Germany	Retrospective Cohort	50	30	Patients were included if they: (1) were in New York Heart Association (NYHA) functional class II or higher because of significant TR at the baseline assessment; (2) received treatment for TR with the MitraClip XTR as monotherapy; (3) had baseline and 30-day clinical (NYHA functional class and/or 6-min walk distance [6MWD]) and echocardiographic evaluation.	Reduction of TR. New York Heart Association functional class improvement, The 6-min walk distance increase.	1.67 ± 0.76
Sugiura et al.	2020	Germany	Retrospective Cohort	44	90	patients who underwent a TTVR using the PASCAL or MitraClipXTR systems.All patients had symptomatic TR and were considered as inoperable or at high-surgical risk. After a standardized diagnostic workup including transesophageal echocardiography (TEE), the decision to perform the intervention was taken by the interdisciplinary heart team.	Reduction in TR severity by at least one grade at 30 days.	0 (5%), 1 (25%), 2 (54%), 3 (16%)
Alet al.	2020	Canada	Prospective Cohort	40	30	Patients with symptomatic severe TR treated with the MitraClip system.	Procedural success, NYHA functional class, TR grade, major adverse cardiac and cerebrovascular events (MACCE) assessed at 30-day follow-up.	NTR (2.3 ± 0.36), XTR (1.8 ± 0.35)
Aurich et al.	2021	Germany	Prospective Cohort	16	30	All patients suffered from symptomatic right-sided heart failure with New York Heart Association functional class III or IV.	postprocedural reduction in TR of at least 1 grade.	1 (38%), 2 (31%), 3 (6%)
Cepas-Guillen et al.	2021	Spain	Prospective Cohort	28	495 (583.5)	Patients who underwent edge-to-edge TTVr	The primary efficacy endpoint was a reduction in the TR of at least one grade. The primary safety endpoint was procedure-related clinical serious adverse events.	1 (64%), 2 (29%), 3 (7%)
Baldus et al.	2022	Europe	Multicenter prospective, single-arm Study	74	30	Patients with severe or greater TR on a 5-grade scale, eligibility to receive treatment with the PASCAL system per the indications for use, and suitability for the procedure as determined by the local heart team.	Proportion of patients with major adverse events (MAEs) at 30 days, Reduction in TR severity as assessed by TEE.	1.8 ± 0.6
Fam et al.	2019	Germany	Nonrandomized, single-arm cohort study	28	30	All patients had heart failure due to severe TR and were deemed at high surgical risk by institutional heart teams.	The primary outcome was procedural success, defined as the implantation of at least 1 device with post-procedural TR grade #2þ, without mortality or conversion to surgery.	NA
Freixa et al.	2022	Spain	Multicenter Retrospective Cohort	34	90	Patients with symptomatic TR.	TR reduction of at least 1 grade assessed by transthoracic echocardiography at discharge.	1 (47%), 2 (44%), 3 (6%), > 3 (3%)
Hellhammer et al.	2022	Germany	Retrospective Cohort	64	NA	Patients underwent transesophageal echocardiography during transcatheter edge-to-edge tricuspid valve repair.	Transesophageal echocardiography related complications.	Non-Complicated (1.6 ± 0.7), Complicated (1.5 ± 0.7)
kalbacher et al.	2017	Germany	Prospective Cohort	766	395 (43.1)	Surgical high-risk patients undergoing MitraClip implantation.	In-hospital, one-year mortality, death, myocardial infarction ± stroke	No/mild TR (1.4 ± 0.6), Moderate TR (1.5 ± 0.6), Severe TR (1.6 ± 0.7)
karam et al.	2019	Germany	Retrospective Cohort	126	187.75 (8.9)	Patients were referred to TTVR if they presented with severe right-sided heart failure (New York Heart Association [NYHA] functional class III to IV despite optimal medical therapy and were deemed inoperable by the heart team.	Renal and liver function improvement.	2.1 ± 0.7
Kodali et al.	2021	USA	Multicenter single-arm, non-randomized trial	34	30	Patients with symptomatic TR despite optimal medical therapy.	Freedom from device or procedure-related adverse events [ Time Frame: 30 days ]	0 (15%), 1 (53%), 2 (32%)

### Quality assessment

**Fig. 2:**
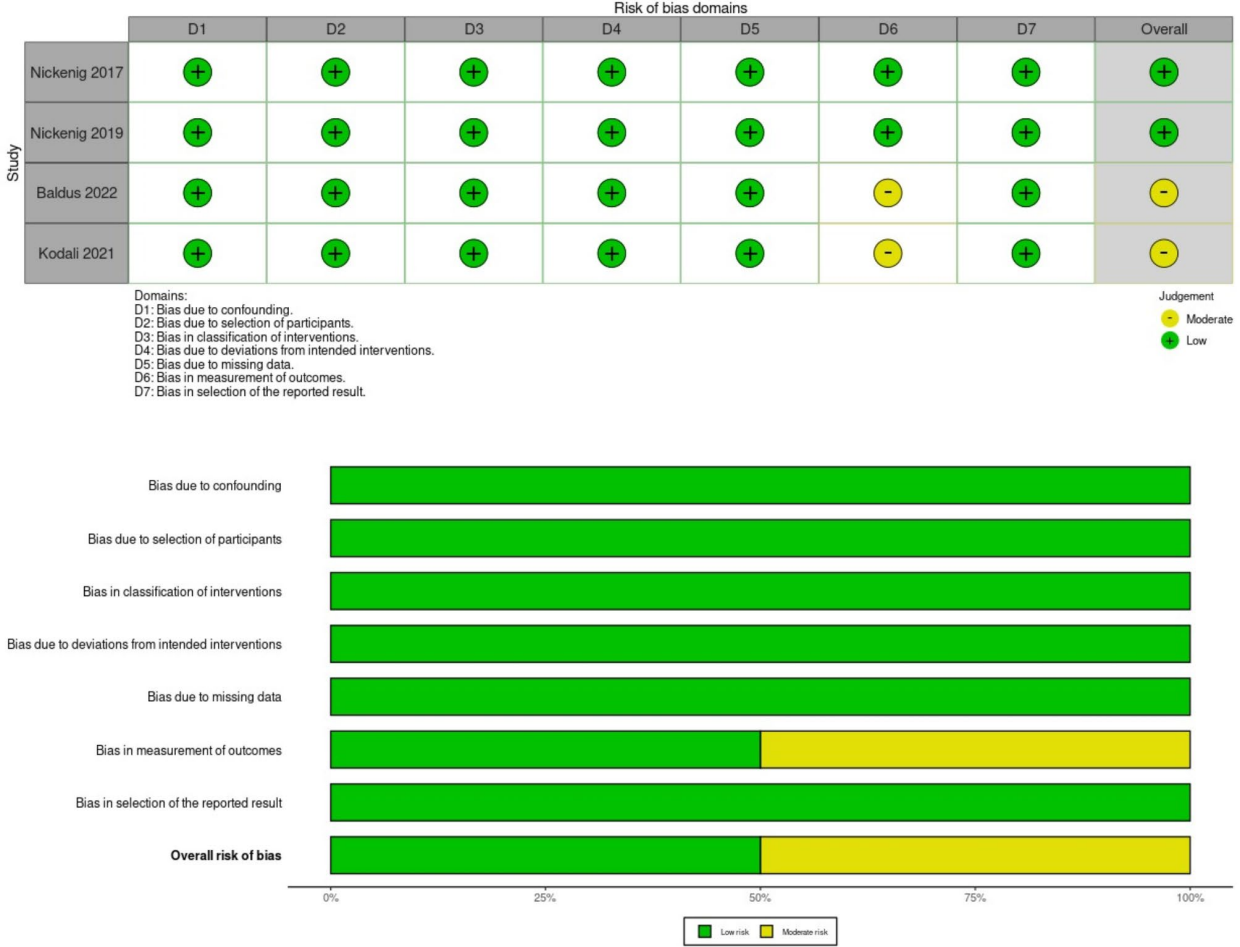
Risk of bias assessment in non-randomized studies using the ROBINS-I tool, showing an overall moderate to high quality of the included studies

According to the Newcastle–Ottawa Scale, all included studies were assessed as poor quality, except for eight studies deemed of good quality. Detailed quality assessment information is available in Supplementary 3 Table S2. According to the ROBINS-I tool, only the studies by Baldus et al. 2022 [32] and Kodali et al. 2021 [23] were identified as having a moderate risk regarding the measurement of outcomes. (Fig. 2) (Supplementary 3 Table S3).

### Primary outcomes

#### Vena contracta width (mm)

**Fig. 3:**
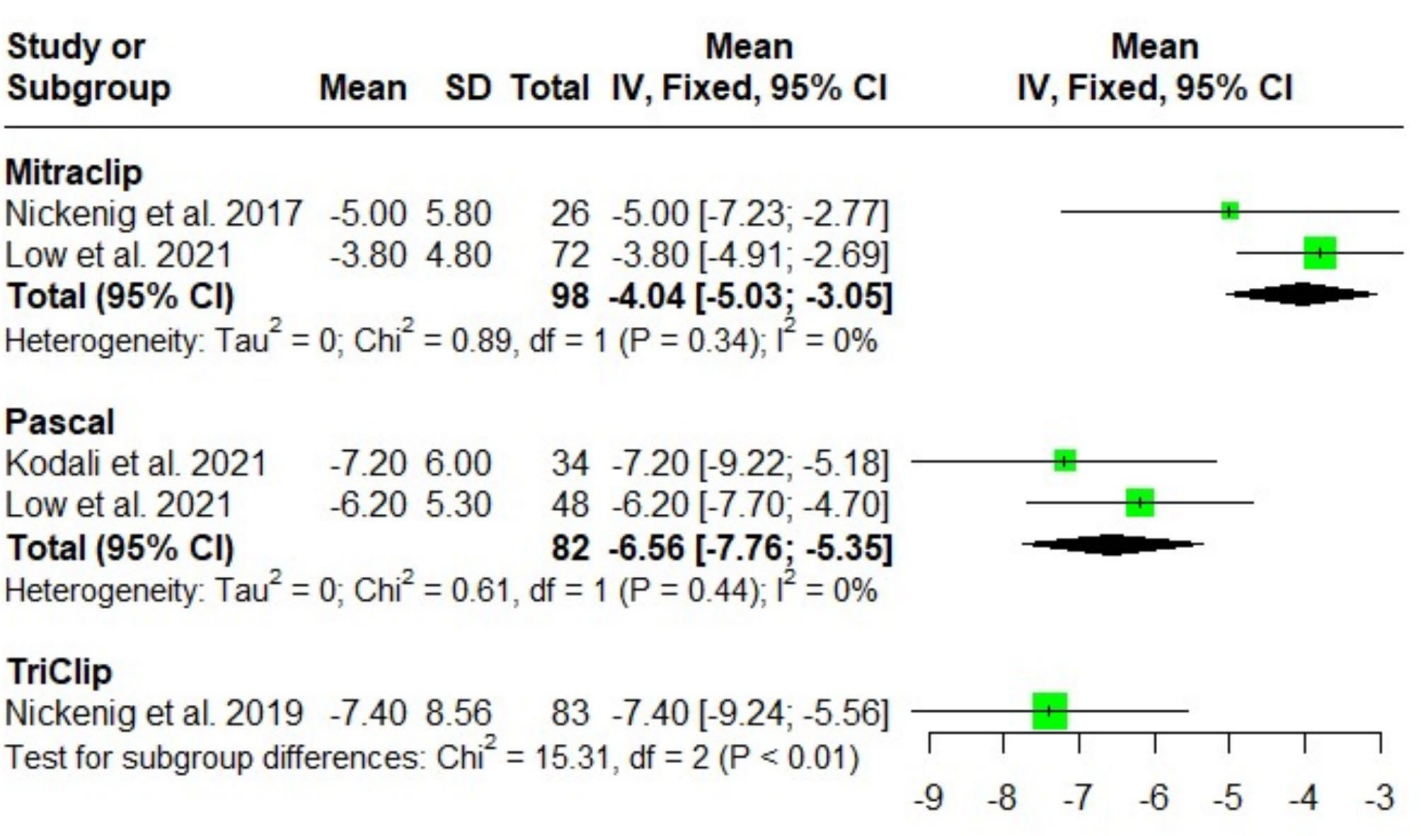
Forest plot of the single-arm meta-analysis of the mean difference (MD) in vena contracta width (mm). The results show a significant reduction in vena contracta width favoring the TriClip system, indicating its effectiveness in reducing tricuspid regurgitation

TriClip demonstrated a more substantial reduction in vena contracta width at discharge compared to both MitraClip and PASCAL [(MD = -7.4; 95%CI: -9.24, -5.56; I^2^ = NA), (MD = -4.04; 95%CI: -5.03, -3.05; I^2^ = 0%), and (MD = -6.56; 95%CI: -7.76, -5.35; I^2^ = 0%); respectively]. Test for subgroup difference showed a significant difference among the three groups (*P* < 0.01) favoring TriClip > Pascal > MitraClip. (Fig. 3).

#### Effective regurgitant orifice area (EROA) (mm²)

**Fig. 4:**
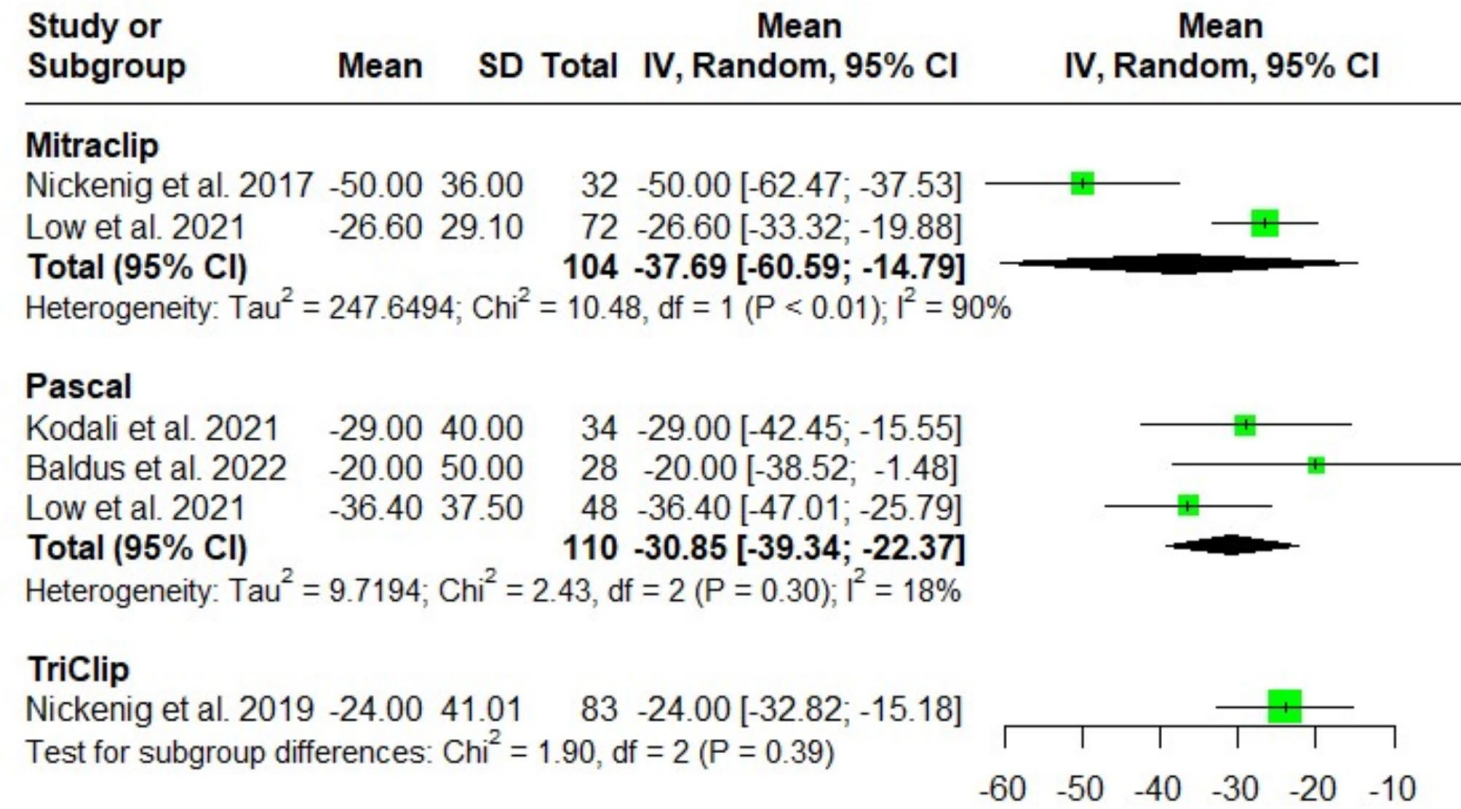
Forest plot of the single-arm meta-analysis of the mean difference (MD) in effective regurgitant orifice area (EROA) (cm²). No significant differences are observed between the systems, suggesting comparable outcomes in EROA reduction

There was no significant difference regarding EROA after MitraClip, Pascal and TriClip at discharge [(MD = -37.69; 95%CI: -60.59, -14.79; I^2^ = 90%), (MD = -30.85; 95%CI: -39.34, -22.37; I^2^ = 18%), and (MD = -24; 95%CI: -32.82, -15.18; I^2^ = NA); respectively]. Test for subgroup difference among the three groups (*P* = 0.39). Following a leave one out sensitivity analysis, the heterogeneity could not be fully resolved due to methodological variations between the pooled studies (Fig. 4).

#### Tricuspid regurgitant volume (ml)

**Fig. 5:**
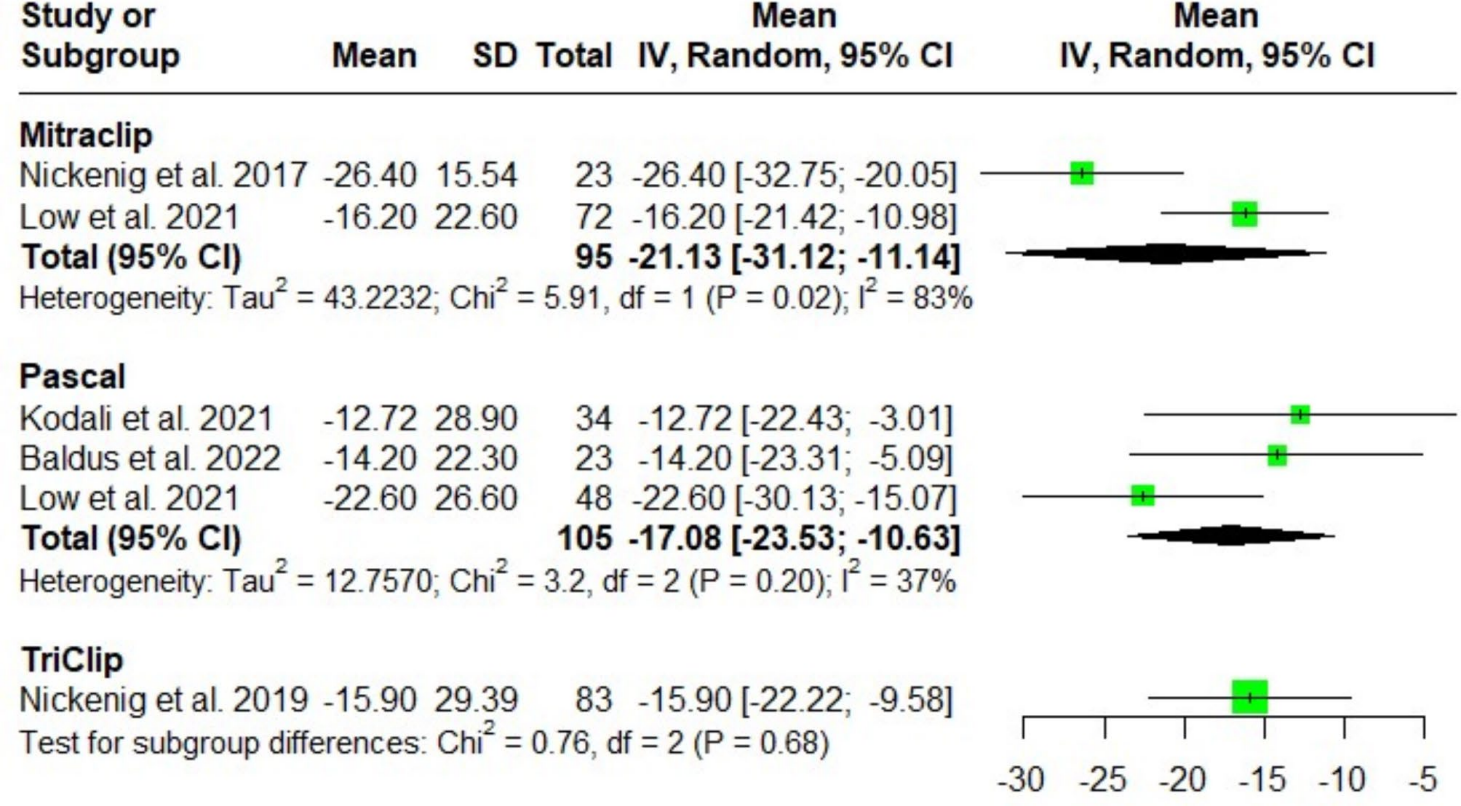
Forest plot of the single-arm meta-analysis of the mean difference (MD) in tricuspid regurgitant volume (ml). The analysis reveals no significant differences between the systems, indicating uniform effects on tricuspid regurgitant volume

Our analysis showed no significant difference regarding the tricuspid regurgitant volume after MitraClip, Pascal and TriClip at discharge [(MD = -21.13; 95%CI: -31.12, -11.14; I^2^ = 83%), (MD = -17.08; 95%CI: -23.53, -10.63; I^2^ = 37%), and (MD = -15.90; 95%CI: -22.22, -9.58; I^2^ = NA); respectively]. Test for subgroup difference showed no significant difference among the three groups (*P* = 0.68). After sensitivity analysis was done, the heterogeneity could not be fully resolved due to methodological variations between the pooled studies. (Fig. 5).

#### Tricuspid annular plane systolic excursion (TAPSE) (mm)

**Fig. 6:**
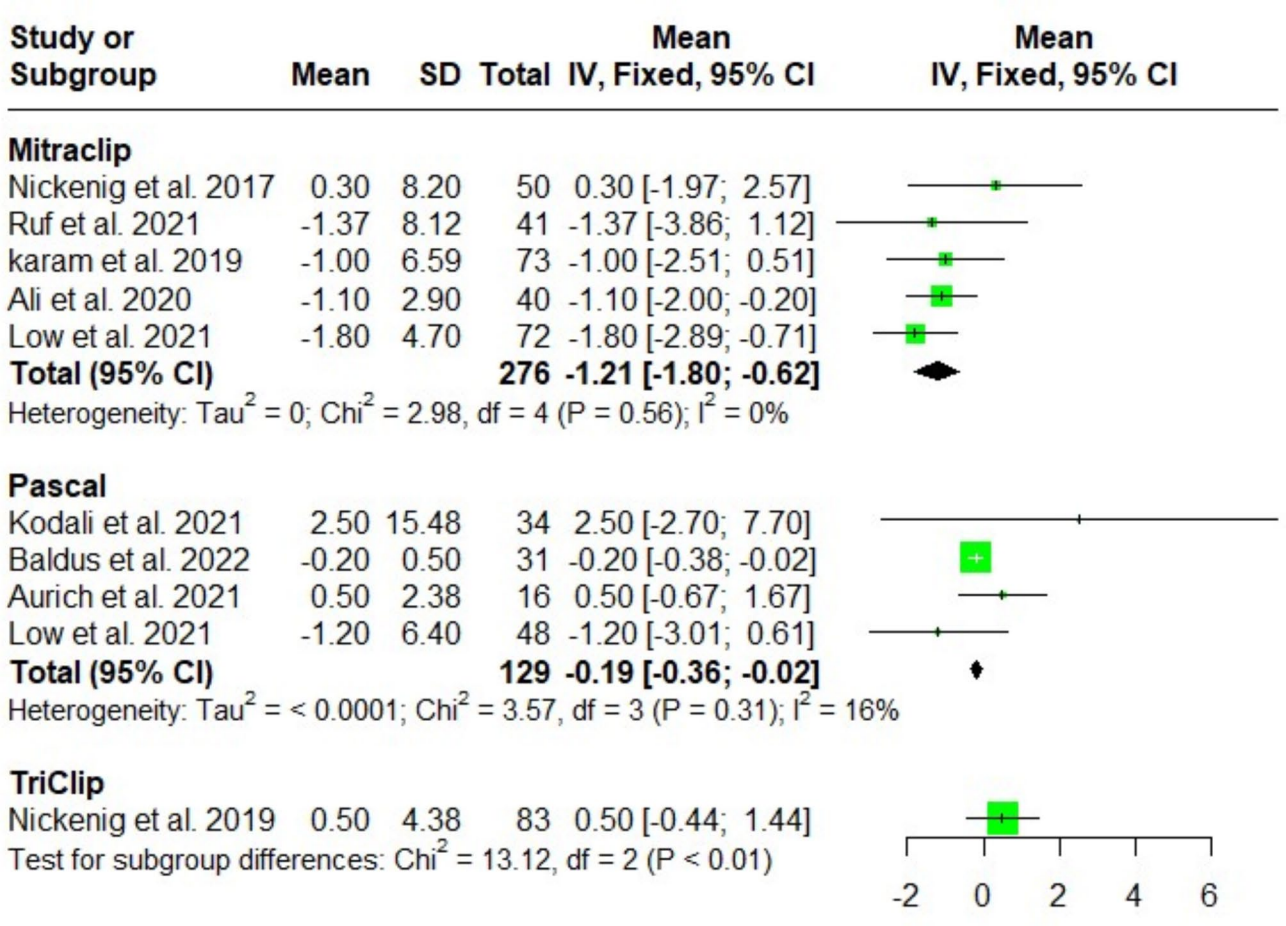
Forest plot of the single-arm meta-analysis of the mean difference (MD) in tricuspid annular plane systolic excursion (TAPSE) (mm). The results demonstrate a significant reduction with the MitraClip system, suggesting improved systolic function

The changes in TAPSE after MitraClip, Pascal, and TriClip procedures at discharge were comparable, with no significant differences [MD = -1.21; 95%CI: -1.80, -0.62; I^2^ = 0%), (MD = -0.19; 95%CI: -0.36, -0.02; I^2^ = 16%), and (MD = -0.5; 95%CI: -0.44, 1.44; I^2^ = NA); respectively]. Test for subgroup differences showed a significant difference among the three groups (*P* < 0.01) (Fig. 6).

#### Right ventricular fractional area change (RV-FAC) (%)

**Fig. 7:**
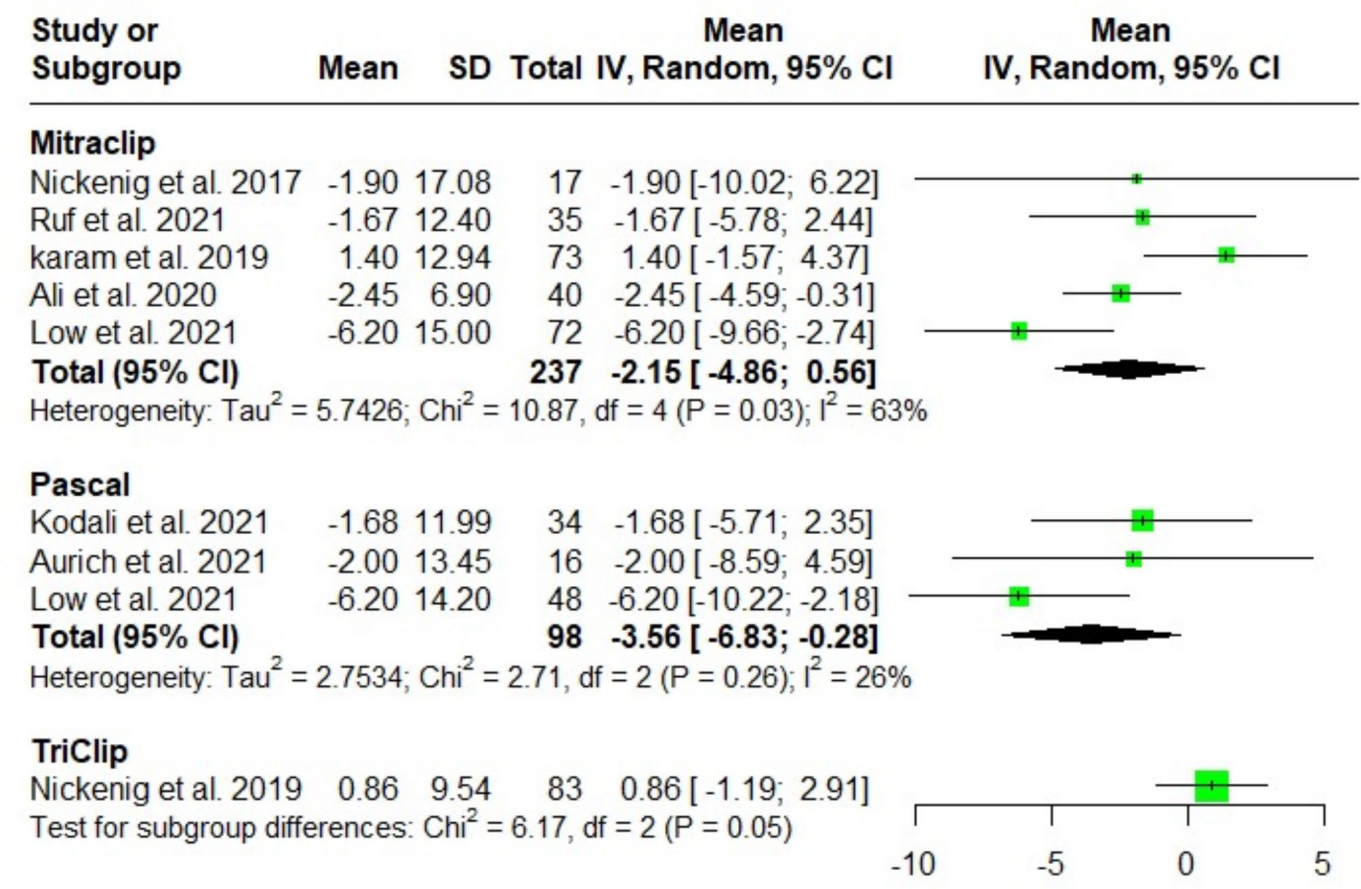
Forest plot of the single-arm meta-analysis of the mean difference (MD) in right ventricular area change (%). No significant differences are observed between the systems, indicating similar effects on right ventricular dimensions

The meta-analysis showed no significant difference in RV-FAC after MitraClip, Pascal and TriClip at discharge [MD = -2.15; 95%CI: -4.86, 0.56; I^2^ = 63%), (MD = -3.56; 95%CI: -6.83, -0.28; I^2^ = 26%), and (MD = 0.86; 95%CI: -1.19, 2.91; I^2^ = NA); respectively]. The test for subgroup differences among the three groups revealed no significant difference (*P* = 0.05). Following a leave one out sensitivity analysis, the heterogeneity could not be fully resolved due to methodological variations between the pooled studies (Fig. 7).

#### Left ventricular ejection fraction (LVEF) (%)

**Fig. 8:**
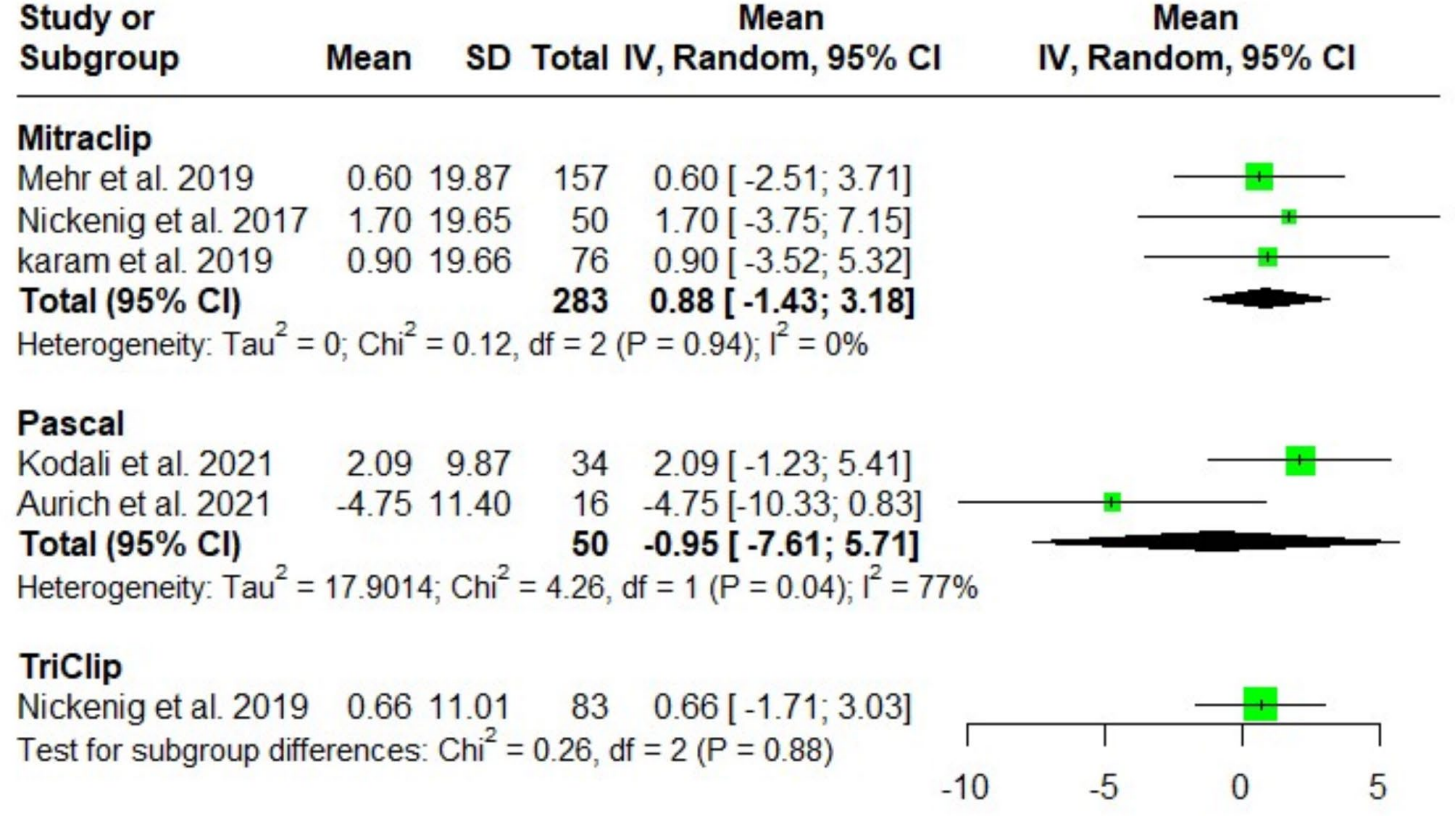
Forest plot of the single-arm meta-analysis of the mean difference (MD) in ejection fraction (%). The analysis shows no significant differences between the systems, reflecting comparable impacts on overall cardiac function

No significant difference was detected regarding the postinterventional LVEF following MitraClip, Pascal and TriClip implantation at discharge [(MD = 0.88; 95%CI: -1.43, 3.18; I^2^ = 0%), (MD = -0.95; 95%CI: -7.61, 5.71; I^2^ = 77%), and (MD = 0.66; 95%CI: -1.71, 3.03; I^2^ = NA); respectively]. The test for subgroup differences among the three groups indicated no significant difference (*P* = 0.88) (Fig. 8).

#### Six-minute walking test (6-MWT) (m.)

**Fig. 9:**
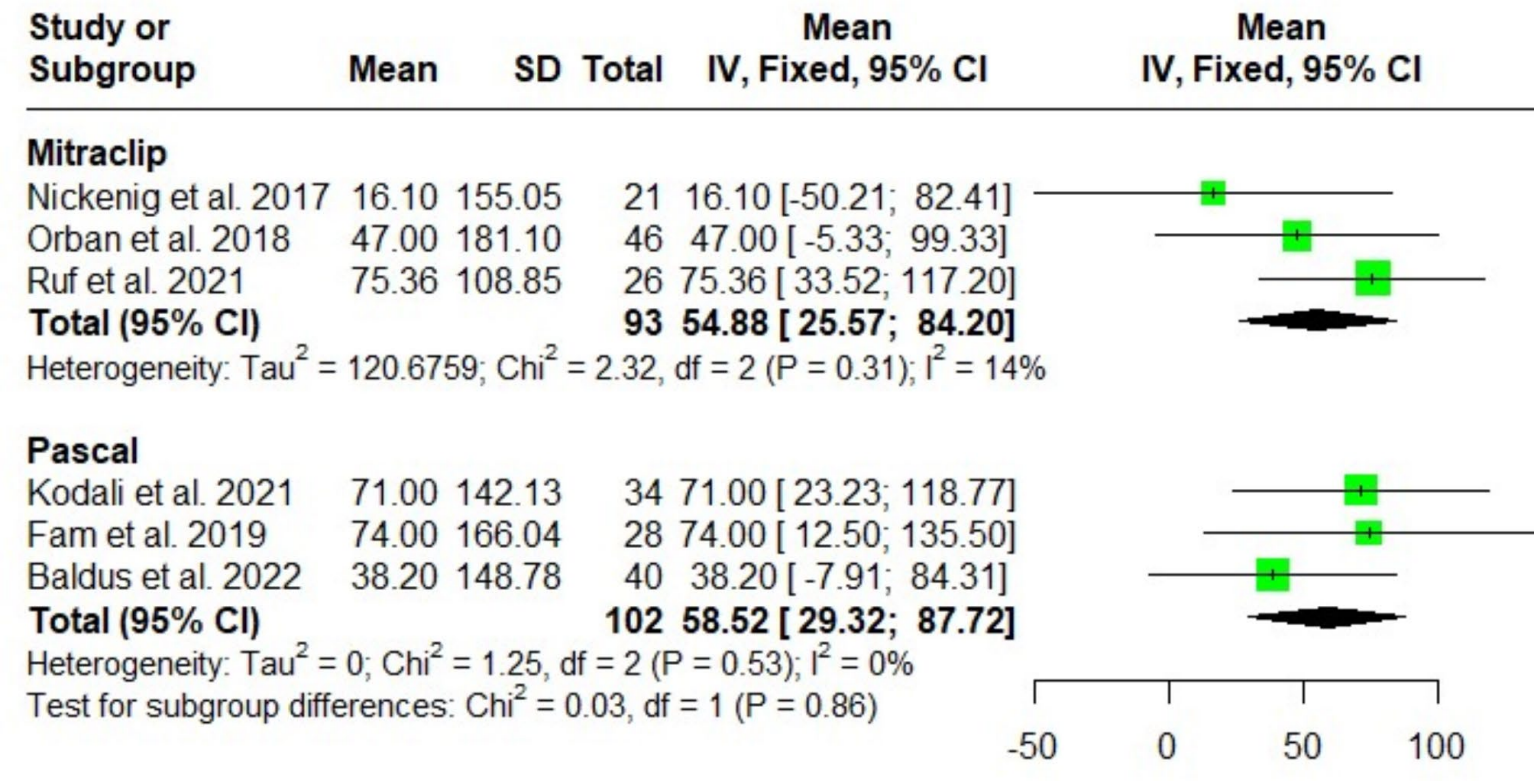
Forest plot of the single-arm meta-analysis of the mean difference (MD) in 6-minute walk test (6-MWT). No significant differences are observed between the systems, indicating similar outcomes in exercise capacity

The change in the six-minute walking test (6-MWT) after MitraClip and Pascal procedures displayed no significant difference at discharge [MD = 54.88; 95%CI: 25.57, 84.20; I^2^ = 14%), and (MD = 58.52; 95%CI: 29.32, 87.72; I^2^ = 0%); respectively]. Test for subgroup differences showed no significant difference among the three groups (*P* = 0.86) (Fig. 9).

### Secondary outcomes

#### Procedural success

Procedural success after MitraClip, Pascal and TriClip at discharge was [81%; 95%CI: 74%, 86%; I^2^ = 72%), (76%; 95%CI: 67%, 84%; I^2^ = 31%), and (92%; 95%CI: 83%, 97%; I^2^ = NA); respectively]. Test for subgroup differences showed no significant difference among the three groups (*P* = 0.05). After sensitivity analysis was done, the heterogeneity could not be fully resolved due to methodological variations between the pooled studies. (Supplementary 4. Figure S1).

#### Single-leaflet device attachment (SLDA)

SLDAs after MitraClip, Pascal and TriClip at discharge were [8%; 95%CI: 4%, 15%; I^2^ = 1%), (6%; 95%CI: 2%, 16%; I^2^ = 0%), and (7%; 95%CI: 2%, 15%; I^2^ = NA); respectively]. Test for subgroup differences showed no significant difference among the three groups (*P* = 0.86) (Supplementary 4. Figure S2).

#### Postoperative NYHA I and II

Number of patients in NYHA I and II after MitraClip, Pascal and TriClip at discharge was [72%; 95%CI: 64%, 80%; I^2^ = 78%), (75%; 95%CI: 57%, 87%; I^2^ = 68%), and (82%; 95%CI: 75%, 88%; I^2^ = 0%); respectively]. Test for subgroup differences showed no significant difference among the three groups (*P* = 0.15) (Supplementary 4. Figure S3).

#### Postoperative NYHA III and IV

Number of patients in NYHA III and IV after MitraClip, Pascal and TriClip at discharge was [33%; 95%CI: 21%, 47%; I^2^ = 85%), (23%; 95%CI: 12%, 38%; I^2^ = 70%), and (18%; 95%CI: 12%, 25%; I^2^ = 0%); respectively]. Test for subgroup differences showed also no significant difference among the three groups (*P* = 0.1) (Supplementary 4. Figure S4).

#### 30-day Mortality

Mortality after 30 days after MitraClip, and Pascal was [(4%; 95%CI: 3%, 5%; I^2^ = 0%), and (6%; 95%CI: 2%, 19%; I^2^ = 0%); respectively]. Test for subgroup differences showed no significant difference among the three groups (*P* = 0.53) (Supplementary 4. Figure S5).

#### Stroke

Stroke after MitraClip, Pascal and TriClip at discharge was [1%; 95%CI: 0%, 1%; I^2^ = 0%), (2%; 95%CI: 1%, 5%; I^2^ = 0%), and (2%; 95%CI: 0%, 10%; I^2^ = 0%); respectively]. Test for subgroup differences showed no significant difference among the three groups (*P* = 0.31) (Supplementary 4. Figure S6).

#### Postoperative major bleeding

Rate of postoperative major bleeding after MitraClip, Pascal and TriClip was [8%; 95%CI: 5%, 13%; I^2^ = 58%), (5%; 95%CI: 2%, 10%; I^2^ = 0%), and (2%; 95%CI: 0%, 10%; I^2^ = 0%); respectively]. Test for subgroup differences showed insignificant difference among the three groups (*P* = 0.18) (Supplementary 4. Figure S7).

#### Fluoroscopy time (min)

Fluoroscopy time during MitraClip, Pascal and TriClip procedures was [(MD = 18.80; 95%CI: 12.09, 25.51; I^2^ = NA), (MD = 37.35; 95%CI: 31.39, 43.31; I^2^ = 31%), and (MD = 29.60; 95%CI: 26.15, 33.05; I^2^ = 0%); respectively]. Test for subgroup differences showed a significant longer fluoroscopy time with Pascal more than both other systems (*P* < 0.01) (Supplementary 4. Figure S8).

#### Hospital stay duration (day)

Length of hospital stay (LOS) after MitraClip, Pascal and TriClip at discharge was [(M = 10.02; 95%CI: 7.43, 12.61; I^2^ = 90%), (M = 3.v83; 95%CI: 1.29, 6.38; I^2^ = 91%), and (M = 4.98; 95%CI: -0.28, 10.25; I^2^ = 99%); respectively]. Test for subgroup differences showed significant difference among the three groups (*P* < 0.01) (Supplementary 4. Figure S9).

#### Procedural time (min)

Procedural time after MitraClip, Pascal and TriClip was [(MD = 117.04; 95%CI: 88.38, 145.69; I^2^ = 95%), (MD = 84.73; 95%CI: 51.97, 117.49; I^2^ = 92%), and (MD = 127.54; 95%CI: 116.44, 138.65; I^2^ = 24%); respectively]. Test for subgroup differences showed no significant difference among the three groups (*P* = 0.05) (Supplementary 4. Figure S10).

## Discussion

This study compared the clinical outcomes and safety profiles of three TTVR devices: MitraClip, PASCAL, and TriClip. TriClip exhibited the greatest effectiveness in reducing vena contracta width, with PASCAL and MitraClip following in effectiveness. There were no significant differences in effective regurgitant orifice area (EROA) or tricuspid regurgitant volume among the devices. Procedural success rates were consistently high for all three devices, and the incidence of single leaflet device attachments (SLDAs) was comparable across the devices. Additionally, there were no significant differences in mortality, stroke rates, or major bleeding events among the three groups.

No significant differences were observed among the three devices regarding effective regurgitant orifice area (EROA) and tricuspid regurgitant volume, indicating that all three devices offer comparable effectiveness in reducing tricuspid regurgitation (TR). However, both the TriClip and PASCAL devices demonstrated a more pronounced reduction in vena contracta compared to the MitraClip. This suggests that the TriClip and PASCAL may be more effective for patients with larger coaptation gaps. These findings could be due to the inherent structural advantages of the TriClip and PASCAL devices over the MitraClip. Notably, the TriClip is specifically designed for the anatomy of the tricuspid valve, whereas the MitraClip was originally developed for mitral valve repair, potentially influencing their relative effectiveness in treating TR [25]. The PASCAL device is designed with a central spacer that optimizes the maximum span width while minimizing excessive tension on the tricuspid leaflets [23, 32, 36]. Furthermore, the PASCAL device features wider clasps compared to the MitraClip, which may distribute forces more evenly across the tricuspid leaflets. The clasps can be operated either simultaneously or independently, allowing for more precise and effective leaflet insertion [23, 32, 36]. It is worth noting that some of these advanced features are now incorporated into the latest generation of MitraClips, known as the MitraClip G4. The MitraClip G4 includes independent grasping capabilities and broader arms, potentially improving its effectiveness in tricuspid valve procedures [37, 38].

TTVR has demonstrated effectiveness in reducing tricuspid regurgitation (TR). This reduction alleviates right ventricular volume overload, thereby improving right ventricular function and reducing venous congestion, which leads to symptom relief [39]. Our meta-analysis revealed that post-interventional changes in right ventricular function, as assessed by TAPSE and RV-FAC, were similar across the three devices, with no significant differences observed. While there was a slight, nonsignificant inclination favoring TriClip, indicating potential additional benefits for patients with impaired right ventricular function, it’s crucial to acknowledge that these variations may be influenced by differences in study cohorts. Further comparative studies are essential to optimize the selection of individual devices for diverse patient cohorts.

The current meta-analysis underscores the efficacy of TTVR in reducing tricuspid regurgitation (TR) and establishes its strong safety profile. The analysis demonstrates high procedural success rates and minimal occurrences of postoperative major bleeding, stroke, and 30-day mortality following TTVR. These positive outcomes were consistent across all three devices examined, highlighting the broader applicability and reliability of TTVR as a safe and effective intervention for TR.

It is important to acknowledge the limitations of this meta-analysis. Firstly, the included studies exhibited significant variations in study design, patient characteristics, and procedural techniques, which could introduce heterogeneity and potential biases. Secondly, the number of studies available for each valve type was limited, particularly for randomized controlled trials (RCTs), affecting the statistical power and generalizability of the findings. Lastly, variations in the follow-up durations of the included studies may have impacted the assessment of long-term outcomes.

## Conclusions

The TriClip showed more effectiveness than the MitraClip and PASCAL in reducing vena contracta width, a key measure of tricuspid regurgitation (TR). While all devices have similar safety profiles and procedural success rates, the TriClip’s superior performance in this outcome suggests it may be particularly beneficial for severe TR. Further research is needed to confirm these findings and evaluate long-term outcomes.

## Electronic supplementary material

Below is the link to the electronic supplementary material.

Supplementary Material 1

Supplementary Material 2

Supplementary Material 3

Supplementary Material 4

Supplementary Material 5

## Data Availability

Most of the data generated or analyzed during this study are included in the supplementary information files. All additional datasets used or analyzed during the current study are available from the corresponding author on reasonable request.

## Abbreviations

TR: Tricuspid regurgitation
TTVR: Transcatheter tricuspid valve repair
TEE: Transesophageal echocardiography
MR: Mitral regurgitation
NYHA: New York Heart Association
6-MWT: Six minutes walking test
EROA: Effective regurgitant orifice area
TAPSE: Tricuspid annular plane systolic excursion

## References

[1] Hahn RT, Tricuspid Regurgitation. N Engl J Med. 2023;388(20):1876–91. 10.1056/NEJMra2216709. 37195943 10.1056/NEJMra2216709

[2] Topilsky Y, Maltais S, Medina Inojosa J, et al. Burden of Tricuspid Regurgitation in patients diagnosed in the community setting. JACC Cardiovasc Imaging. 2019;12(3):433–42. 10.1016/j.jcmg.2018.06.014. 30121261 10.1016/j.jcmg.2018.06.014

[3] Axtell AL, Bhambhani V, Moonsamy P, et al. Surgery does not improve survival in patients with isolated severe tricuspid regurgitation. J Am Coll Cardiol. 2019;74(6):715–25. 10.1016/j.jacc.2019.04.028. 31071413 10.1016/j.jacc.2019.04.028 PMC8890054

[4] Scotti A, Sturla M, Granada JF, et al. Outcomes of isolated tricuspid valve replacement: a systematic review and meta-analysis of 5,316 patients from 35 studies. EuroIntervention. 2022;18(10):840–51. 10.4244/EIJ-D-22-00442. 36197445 10.4244/EIJ-D-22-00442 PMC10167545

[5] Muntané-Carol G, Taramasso M, Miura M, et al. Transcatheter tricuspid valve intervention in patients with right ventricular dysfunction or pulmonary hypertension: insights from the TriValve Registry. Circ Cardiovasc Interv. 2021;14(2):e009685. 10.1161/CIRCINTERVENTIONS.120.009685. 33541097 10.1161/CIRCINTERVENTIONS.120.009685

[6] Sorajja P, Whisenant B, Hamid N, et al. Transcatheter repair for patients with tricuspid regurgitation. N Engl J Med. 2023;388(20):1833–42. 10.1056/NEJMoa2300525. 36876753 10.1056/NEJMoa2300525

[7] Nickenig G, Kowalski M, Hausleiter J, et al. Transcatheter treatment of severe tricuspid regurgitation with the edge-to-Edge MitraClip technique. Circulation. 2017;135(19):1802–14. 10.1161/CIRCULATIONAHA.116.024848. 28336788 10.1161/CIRCULATIONAHA.116.024848

[8] Taramasso M, Alessandrini H, Latib A, et al. Outcomes after current transcatheter tricuspid valve Intervention: mid-term results from the International TriValve Registry. JACC Cardiovasc Interv. 2019;12(2):155–65. 10.1016/j.jcin.2018.10.022. 30594510 10.1016/j.jcin.2018.10.022

[9] Taramasso M, Benfari G, van der Bijl P, et al. Transcatheter Versus Medical treatment of patients with symptomatic severe tricuspid regurgitation. J Am Coll Cardiol. 2019;74(24):2998–3008. 10.1016/j.jacc.2019.09.028. 31568868 10.1016/j.jacc.2019.09.028

[10] Higgins JP, Thomas J, Chandler J et al. Cochrane handbook for systematic reviews of interventions. Second edition. Hoboken, NJ: Wiley-Blackwell; 2020.

[11] Page MJ, McKenzie JE, Bossuyt PM, et al. The PRISMA 2020 statement: an updated guideline for reporting systematic reviews. BMJ. 2021;372:n71. 10.1136/bmj.n71. 33782057 10.1136/bmj.n71 PMC8005924

[12] Fernandez P. Zotero: information management software 2.0. Libr Hi Tech News. 2011;28(4):5–7. 10.1108/07419051111154758.

[13] Birkinbine BJ et al. Microsoft Corporation. Global Media Giants. In: Birkinbine, Gómez Herausgeber 2019 – Global Media Giants:383–397.

[14] Sterne JA, Hernán MA, Reeves BC, et al. ROBINS-I: a tool for assessing risk of bias in non-randomised studies of interventions. BMJ. 2016;355:i4919. 10.1136/bmj.i4919. 27733354 10.1136/bmj.i4919 PMC5062054

[15] Wells G, Shea B, O’Connell D et al. The Newcastle–Ottawa Scale (NOS) for Assessing the Quality of Non-Randomized Studies in Meta-Analysis. ᅟ. 2000;&#4447.

[16] Hellhammer K, Schueler R, Eißmann M, et al. Safety of transesophageal echocardiography during transcatheter edge-to-edge tricuspid valve repair: a single-center experience. Front Cardiovasc Med. 2022;9:856028. 10.3389/fcvm.2022.856028. 36304534 10.3389/fcvm.2022.856028 PMC9592690

[17] Karam N, Braun D, Mehr M, et al. Impact of transcatheter tricuspid valve repair for severe tricuspid regurgitation on kidney and liver function. JACC Cardiovasc Interv. 2019;12(15):1413–20. 10.1016/j.jcin.2019.04.018. 31126888 10.1016/j.jcin.2019.04.018

[18] Sugiura A, Vogelhuber J, Öztürk C, et al. PASCAL versus MitraClip-XTR edge-to-edge device for the treatment of tricuspid regurgitation: a propensity-matched analysis. Clin Res Cardiol. 2021;110(3):451–9. 10.1007/s00392-020-01784-w. 33313975 10.1007/s00392-020-01784-w PMC7907034

[19] Otto S, Velichkov M, Hamadanchi A, Schulze PC, Moebius-Winkler S. The impact of tricuspid annular geometry on outcome after percutaneous edge-to-edge repair for severe tricuspid regurgitation. Cardiol J. 2021;28(4):579–88. 10.5603/CJ.a2021.0046. 33942279 10.5603/CJ.a2021.0046 PMC8277011

[20] Ohno Y, Attizzani GF, Capodanno D, et al. Association of tricuspid regurgitation with clinical and echocardiographic outcomes after percutaneous mitral valve repair with the MitraClip System: 30-day and 12-month follow-up from the GRASP Registry. Eur Heart J Cardiovasc Imaging. 2014;15(11):1246–55. 10.1093/ehjci/jeu114. 24939944 10.1093/ehjci/jeu114

[21] Mehr M, Taramasso M, Besler C, et al. 1-Year outcomes after edge-to-edge valve repair for symptomatic tricuspid regurgitation: results from the TriValve Registry. JACC Cardiovasc Interv. 2019;12(15):1451–61. 10.1016/j.jcin.2019.04.019. 31395215 10.1016/j.jcin.2019.04.019

[22] Löw K, Orban M, Stocker T, et al. Acute and short-term results of MitraClip XTR vs. PASCAL Transcatheter Valve Repair System for Edge-to-edge repair of severe tricuspid regurgitation. Struct Heart. 2021;5(5):510–7. 10.1080/24748706.2021.1954272.

[23] Kodali S, Hahn RT, Eleid MF, et al. Feasibility study of the transcatheter valve repair system for severe tricuspid regurgitation. J Am Coll Cardiol. 2021;77(4):345–56. 10.1016/j.jacc.2020.11.047. 33509390 10.1016/j.jacc.2020.11.047

[24] Mahowald MK, Nishimura RA, Pislaru SV, et al. Reduction in right atrial pressures is Associated with hemodynamic improvements after transcatheter edge-to-edge repair of the tricuspid valve. Circ Cardiovasc Interv. 2021;14(12):e010557. 10.1161/CIRCINTERVENTIONS.121.010557. 34814697 10.1161/CIRCINTERVENTIONS.121.010557

[25] Nickenig G, Weber M, Lurz P, et al. Transcatheter edge-to-edge repair for reduction of tricuspid regurgitation: 6-month outcomes of the TRILUMINATE single-arm study. Lancet. 2019;394(10213):2002–11. 10.1016/S0140-6736(19)32600-5. 31708188 10.1016/S0140-6736(19)32600-5

[26] Orban M, Besler C, Braun D, et al. Six-month outcome after transcatheter edge-to-edge repair of severe tricuspid regurgitation in patients with heart failure. Eur J Heart Fail. 2018;20(6):1055–62. 10.1002/ejhf.1147. 29405554 10.1002/ejhf.1147

[27] Ruf TF, Hahn RT, Kreidel F, et al. Short-term clinical outcomes of transcatheter tricuspid valve repair with the Third-Generation MitraClip XTR System. JACC Cardiovasc Interv. 2021;14(11):1231–40. 10.1016/j.jcin.2021.03.033. 34112460 10.1016/j.jcin.2021.03.033

[28] Toyama K, Ayabe K, Kar S, et al. Postprocedural changes of Tricuspid Regurgitation after MitraClip Therapy for Mitral Regurgitation. Am J Cardiol. 2017;120(5):857–61. 10.1016/j.amjcard.2017.05.044. 28689751 10.1016/j.amjcard.2017.05.044

[29] Kalbacher D, Schäfer U, von Bardeleben RS, et al. Impact of tricuspid valve regurgitation in surgical high-risk patients undergoing MitraClip implantation: results from the TRAMI registry. EuroIntervention. 2017;12(15):e1809–16. 10.4244/EIJ-D-16-00850. 28089952 10.4244/EIJ-D-16-00850

[30] Freixa X, Arzamendi D, Del Trigo M, et al. The TriClip system for edge-to-edge transcatheter tricuspid valve repair. A Spanish multicenter study. Rev Esp Cardiol (Engl Ed). 2022;75(10):797–804. 10.1016/j.rec.2022.01.007. 35288060 10.1016/j.rec.2022.01.007

[31] Aurich M, Volz MJ, Mereles D, et al. Initial experience with the PASCAL Ace Implant System for treatment of severe tricuspid regurgitation. Circ Cardiovasc Interv. 2021;14(9):e010770. 10.1161/CIRCINTERVENTIONS.121.010770. 34433291 10.1161/CIRCINTERVENTIONS.121.010770

[32] Baldus S, Schofer N, Hausleiter J, et al. Transcatheter valve repair of tricuspid regurgitation with the PASCAL system: TriCLASP study 30-day results. Catheter Cardiovasc Interv. 2022;100(7):1291–9. 10.1002/ccd.30450. 36378678 10.1002/ccd.30450

[33] Cepas-Guillen PL, La Fuente Mancera JC, Bofarull G. Initial results after the implementation of an Edge-To-Edge transcatheter tricuspid valve repair program. J Clin Med. 2021;10(18). 10.3390/jcm10184252. 10.3390/jcm10184252 PMC8471561 34575362

[34] Ali FM, Ong G, Edwards J, Connelly KA, Fam NP. Comparison of transcatheter tricuspid valve repair using the MitraClip NTR and XTR systems. Int J Cardiol. 2021;327:156–62. 10.1016/j.ijcard.2020.11.073. 33301831 10.1016/j.ijcard.2020.11.073

[35] Fam NP, Braun D, von Bardeleben RS, et al. Compassionate use of the PASCAL Transcatheter Valve Repair System for severe tricuspid regurgitation: a Multicenter, Observational, First-in-human experience. JACC Cardiovasc Interv. 2019;12(24):2488–95. 10.1016/j.jcin.2019.09.046. 31857018 10.1016/j.jcin.2019.09.046

[36] Lim DS, Smith RL, Gillam LD, et al. Randomized comparison of transcatheter edge-to-edge repair for degenerative mitral regurgitation in Prohibitive Surgical Risk patients. JACC Cardiovasc Interv. 2022;15(24):2523–36. 10.1016/j.jcin.2022.09.005. 36121247 10.1016/j.jcin.2022.09.005

[37] Ives CW, Prejean SP, Vardas PN, von Mering G, Ahmed MI. Initial experiences with the MitraClip G4: review of the Novel device features. Innovations (Phila). 2021;16(5):448–55. 10.1177/15569845211030862. 34420410 10.1177/15569845211030862

[38] Chakravarty T, Makar M, Patel D, et al. Transcatheter edge-to-edge mitral valve repair with the MitraClip G4 System. JACC Cardiovasc Interv. 2020;13(20):2402–14. 10.1016/j.jcin.2020.06.053. 33011141 10.1016/j.jcin.2020.06.053

[39] Lurz P, von Stephan R, Weber M, et al. Transcatheter edge-to-edge repair for treatment of Tricuspid Regurgitation. J Am Coll Cardiol. 2021;77(3):229–39. 10.1016/j.jacc.2020.11.038. 33478646 10.1016/j.jacc.2020.11.038

