# Supplementary material for: Comparative analysis of MitraClip/TriClip and PASCAL in transcatheter tricuspid valve repair for tricuspid regurgitation: a systematic review and meta-analysis
Source: BMC Cardiovasc Disord. 2024 Oct 14;24:557. doi: 10.1186/s12872-024-04201-6 (PMC11476464; doi:10.1186/s12872-024-04201-6)
Supplement: Supplementary file 1 — Supplementary Material 1 [file 12872_2024_4201_MOESM1_ESM.docx]

**Supplementary Legend**

- **Supplementary 1.** Search Strategy for literature search in each database.
- **Supplementary 2.** Baseline characteristics table of the included studies.
  - ***Table S1.*** Baseline Characteristics of the included studies
- **Supplementary 3.** Risk of bias assessment of the included study designs.
  - ***Table S2*** . Quality assessment criteria used for Cohort Studies.
  - ***Table S3***. ROBINS-I quality assessment for non-randomized studies of intervention.
- **Supplementary 4.** Analysis figures (Forest plot of the secondary outcomes single arm meta-analysis).
  - ***Figure S1.*** Forest plot of the single arm meta-analysis of risk ratio (RR) in procedural success.
  - ***Figure S2.*** Forest plot of the single arm meta-analysis of risk ratio (RR) in single-leaflet device attachment.
  - ***Figure S3.*** Forest plot of the single arm meta-analysis of risk ratio (RR) in postoperative NYHA 1 or 2.
  - ***Figure S4.*** Forest plot of the single arm meta-analysis of risk ratio (RR) in postoperative NYHA 3-4.
  - ***Figure S5.*** Forest plot of the single arm meta-analysis of risk ratio (RR) in 30-day Mortality.
  - ***Figure S6.*** Forest plot of the single arm meta-analysis of risk ratio (RR) in stroke.
  - ***Figure S7.*** Forest plot of the single arm meta-analysis of risk ratio (RR) in major bleeding.
  - ***Figure S8.*** Forest plot of the single arm meta-analysis of mean difference (MD) in fluoroscopy time (min).
  - ***Figure S9.*** Forest plot of the single arm meta-analysis of mean difference (MD) in hospital stay (Day).
  - ***Figure 10.*** Forest plot of the single arm meta-analysis of mean difference (MD) in procedural Time (min).
