# Supplementary material for: Comparative analysis of MitraClip/TriClip and PASCAL in transcatheter tricuspid valve repair for tricuspid regurgitation: a systematic review and meta-analysis
Source: BMC Cardiovasc Disord. 2024 Oct 14;24:557. doi: 10.1186/s12872-024-04201-6 (PMC11476464; doi:10.1186/s12872-024-04201-6)
Supplement: Supplementary file 2 — Supplementary Material 2 [file 12872_2024_4201_MOESM2_ESM.docx]

**Supplementary 1.** Search Strategy for each database.

**Search strategy**: (Tricuspid) AND (Insufficiency OR Incompetence OR Regurgitation) AND (MitraClip) OR (Pascal) OR (TriClip)

**PubMed**

Strategy: (Tricuspid) AND (Insufficiency OR Incompetence OR Regurgitation) AND (MitraClip OR Pascal OR TriClip)

Results: 201

**Cochrane**

Strategy: (Tricuspid) AND (Insufficiency OR Incompetence OR Regurgitation) AND (MitraClip OR Pascal OR TriClip)

Results: 873

**WOS**

Strategy: (Tricuspid) AND (Insufficiency OR Incompetence OR Regurgitation) AND (MitraClip OR Pascal OR TriClip)

Results: 305

**SCOPUS**

Strategy: (Tricuspid) AND (Insufficiency OR Incompetence OR Regurgitation) AND (MitraClip OR Pascal OR TriClip)

Results: 563

**Medline (Ovid)**

Strategy: (Tricuspid) AND (Insufficiency OR Incompetence OR Regurgitation) AND (MitraClip OR Pascal OR TriClip)

Results: 251

**Date of search:** 4/23/2023

**Total**: 2239

**After removing the duplicate**: 1505

**After title and abstract screening**: 129

**After full text**: 21
