# Supplementary material for: Comparative analysis of MitraClip/TriClip and PASCAL in transcatheter tricuspid valve repair for tricuspid regurgitation: a systematic review and meta-analysis
Source: BMC Cardiovasc Disord. 2024 Oct 14;24:557. doi: 10.1186/s12872-024-04201-6 (PMC11476464; doi:10.1186/s12872-024-04201-6)

**Supplementary 4. Analysis figures for secondary outcomes**

**Supplementary Figures**

**Figure S1.** Forest plot of the single arm meta-analysis of risk ratio (RR) in procedural success.


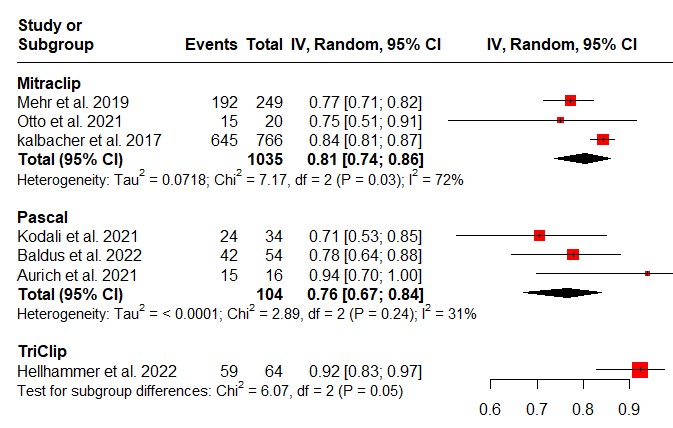


**Figure S2.** Forest plot of the single arm meta-analysis of risk ratio (RR) in single-leaflet device attachment.


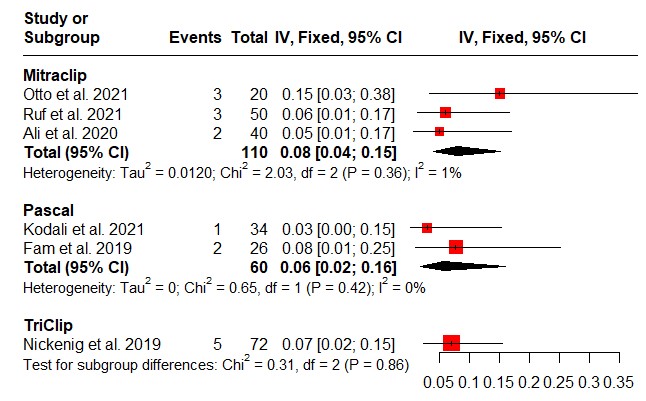


**Figure S3.** Forest plot of the single arm meta-analysis of risk ratio (RR) in postoperative NYHA 1 or 2


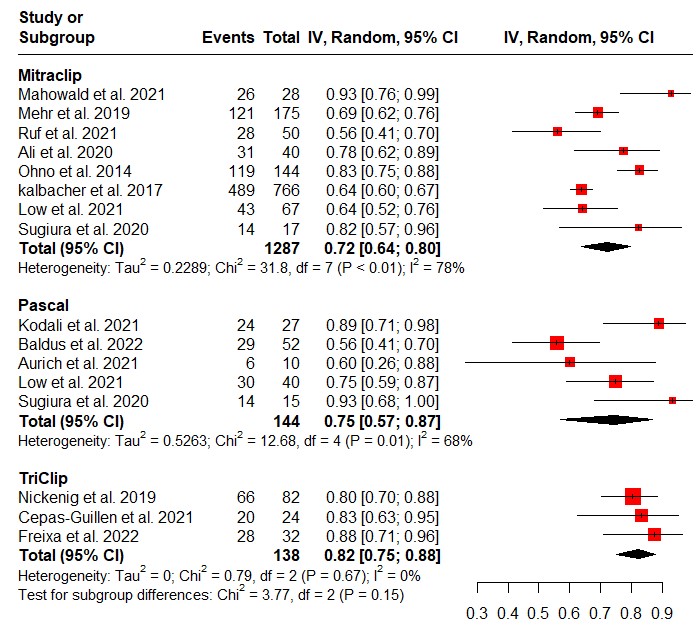


**Figure S4.** Forest plot of the single arm meta-analysis of risk ratio (RR) in postoperative NYHA 3-4.


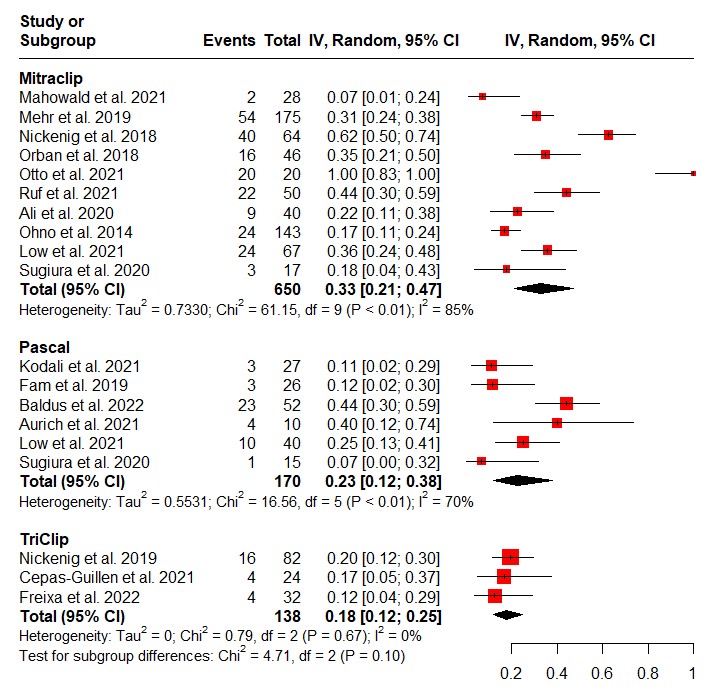


**Figure S5.** Forest plot of the single arm meta-analysis of risk ratio (RR) in 30-day Mortality.


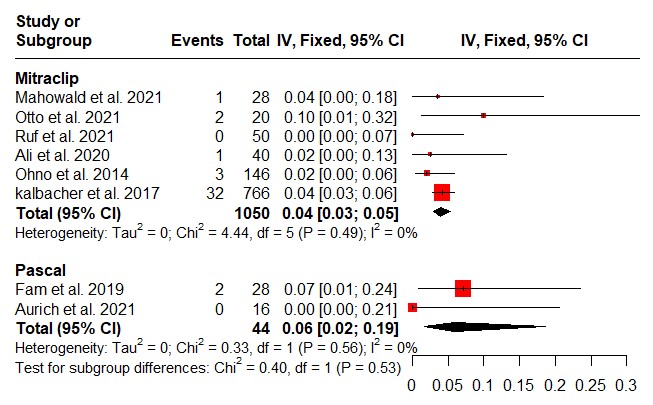


**Figure S6.** Forest plot of the single arm meta-analysis of risk ratio (RR) in stroke


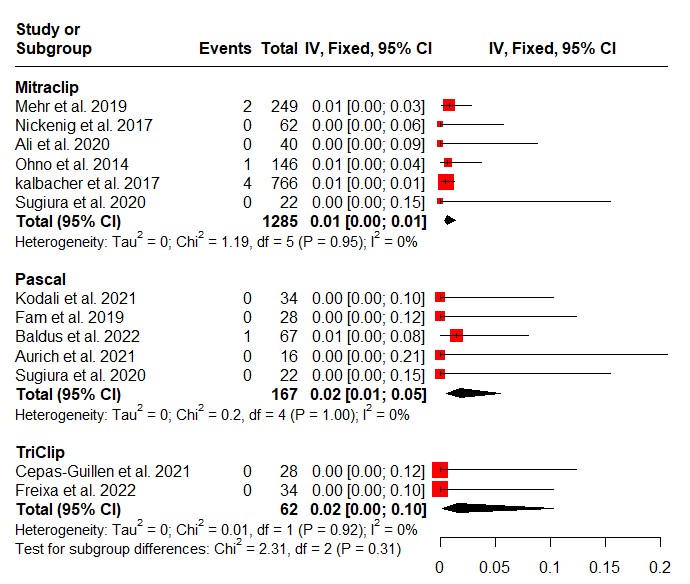


**Figure S7.** Forest plot of the single arm meta-analysis of risk ratio (RR) in major bleeding


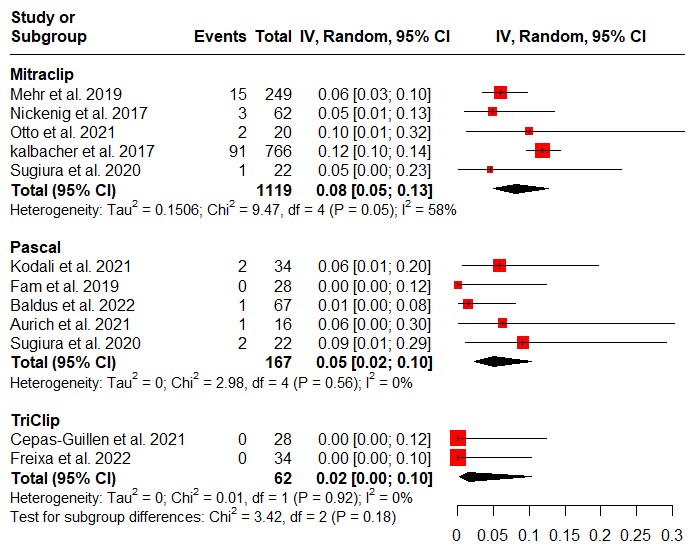


**Figure S8.** Forest plot of the single arm meta-analysis of mean difference (MD) in fluoroscopy time (min)


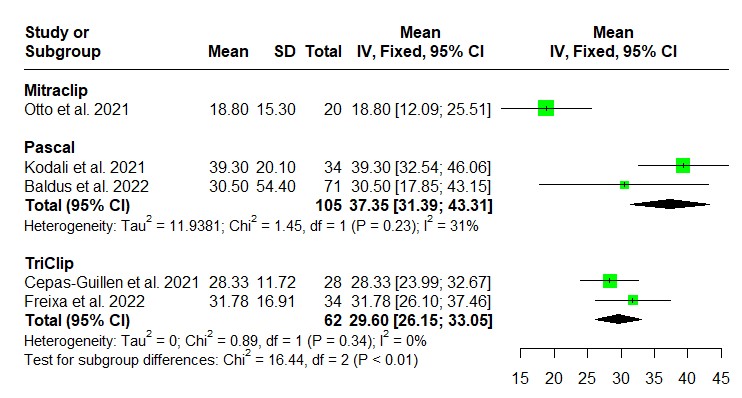


**Figure S9.** Forest plot of the single arm meta-analysis of mean difference (MD) in hospital stay (Day).


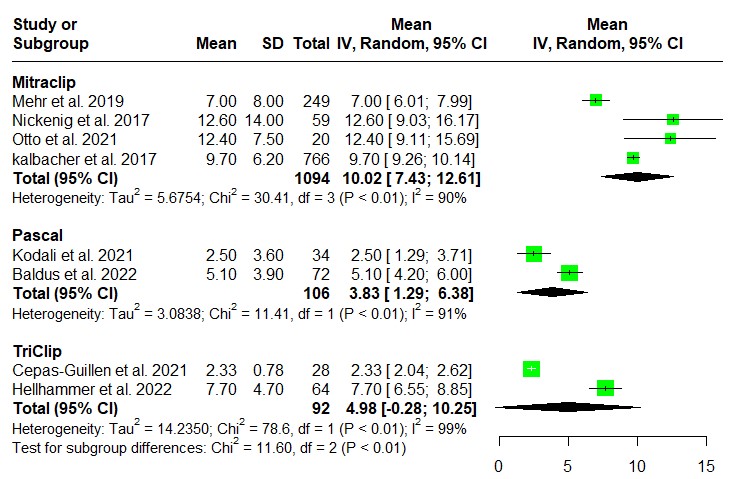


**Figure S10.** Forest plot of the single arm meta-analysis of mean difference (MD) in procedural Time (min).


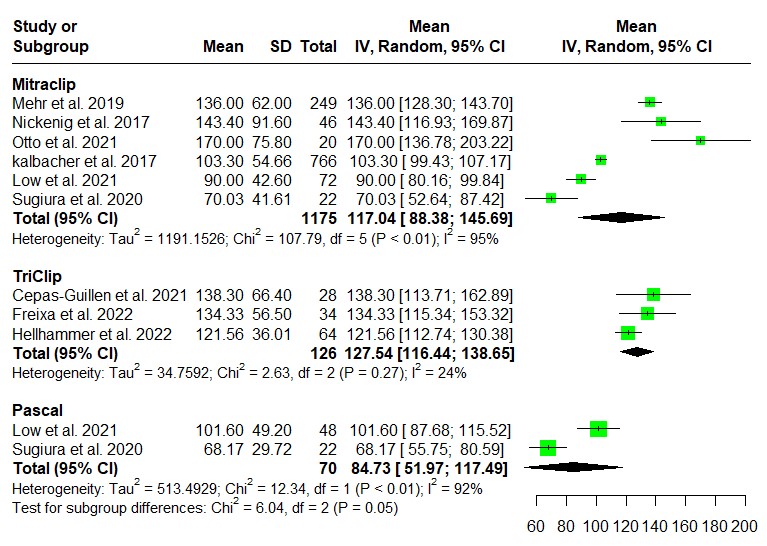

Supplement: Supplementary file 5 — Supplementary Material 5 [file 12872_2024_4201_MOESM5_ESM.docx]
